# Mapping *Aspergillus niger* Metabolite Biomarkers for In Situ and Early Evaluation of Table Grapes Contamination

**DOI:** 10.3390/foods10112870

**Published:** 2021-11-19

**Authors:** Joao Raul Belinato, Carina Pedrosa Costa, Adelaide Almeida, Silvia M. Rocha, Fabio Augusto

**Affiliations:** 1 Institute of Chemistry, University of Campinas and National Institute of Science and Technology in Bioanalysis (INCTBio), Campinas 13083-970, Brazil; joaoraul16@gmail.com; 2 Department of Chemistry & LAQV-REQUIMTE, University of Aveiro, 3810-193 Aveiro, Portugal; carina.pedrosa@ua.pt; 3 Department of Biology & CESAM, University of Aveiro, 3810-193 Aveiro, Portugal; aalmeida@ua.pt

**Keywords:** *Aspergillus niger*, food contamination, GC×GC, metabolites, HS-SPME

## Abstract

The *Aspergillus niger* exometabolome was recently investigated using advanced gas chromatography in tandem with multivariate analysis, which allowed a metabolite biomarker pattern to be proposed. Microbial metabolomics patterns have gained enormous relevance, mainly due to the amount of information made available, which may be useful in countless processes. One of the great challenges in microbial metabolomics is related to applications in more complex systems of metabolomics information obtained from studies carried out in culture media, as complications may occur due to the dynamic nature of biological systems. Thus, the main objective of this research was to evaluate the applicability of the *A. niger* metabololite biomarkers pattern for in situ and early evaluation of table grapes contamination, used as study model. *A. niger* is a ubiquitous fungus responsible for food contamination, being reported as one of the main agents of the black mold disease, a serious post-harvest pathology of table grapes. This work included analysis from 1 day of growth time of pure *A. niger* cultures, *A. niger* cultures obtained from previously contaminated grapes, and finally, an in situ solid-phase microextraction (SPME) approach directly on previously contaminated table grapes. Supervised multivariate analysis was performed which revealed that after 1 day of inoculation it was possible to detect *A. niger* biomarkers, which can be extremely useful in making this type of method possible for the rapid detection of food contamination. The results obtained confirm the potential applicability of the pattern of *A. niger* biomarkers for early detection of the fungi (after 1 day of contamination), and may be further explored for access food susceptibility to fungi contamination, based on direct analysis of the food item.

## 1. Introduction

Fungal contamination plays a major role in food spoilage and represents a problem which can result in huge economic losses, deterioration of food quality, reduction in nutrients availability, and contamination with compounds with high potential toxicity, such as mycotoxins [1]. Mycotoxins are secondary metabolites produced by fungi that have adverse health effects on humans, animals, and crops. In this sense, prevention and control of these toxigenic fungi and mycotoxins in agricultural commodities have been priority objectives in food quality and safety [2,3,4]. Among different classes of food which are susceptible to fungal infection, table grapes are a very common case due to their thin pericarp and succulent flesh. Thus, grapes can be easily contaminated with filamentous fungi in different steps across their production chain and for this reason, the quality control of table grapes must be very strict and effective [5,6].

Some of the most relevant species related to the fungal infection of grapes are members of the genus Aspergillus [5]. *Aspergillus niger* is one of the main species responsible for contamination in grapes and derived products, causing serious economic losses, with the distribution of black aspergilli in table grapes reported worldwide [6,7,8]. The production of mycotoxins by *A. niger* is well known but the contribution to mycotoxin content in foodstuffs as well as the differences in production among species are controversial topics. Ochratoxin A is one of the metabolites usually associated to contamination by *A. niger* [2,9,10,11], including in grapes [7]. However, as it is not specific for this species, it therefore cannot be used as an unambiguous biomarker. On the other hand, recent studies reported a set of metabolites that may be assigned as *A. niger* biomarkers [12,13,14]. These metabolites are distributed in several chemical families, such as aldehydes, ethers, alcohols, esters, ketones, hydrocarbons, and terpenic compounds. Metabolic pathways such as amino acid metabolism, biosynthesis and metabolism of fatty acids, degradation of aromatic compounds, mono and sesquiterpenoid synthesis, and carotenoid cleavage are found to be related to these set of compounds [14]. Nevertheless, studies on production of these metabolites specifically by *A. niger* have still not been completely explored [14,15].

Considering the importance of early diagnosis in food authentication and contamination, metabolomics enables the development of rapid and highly accurate chemical methods for the identification of the species [9,16,17,18,19,20,21,22]. Microbial metabolomics represents a holistic approach for comprehensive monitoring of metabolites directly linked to cellular metabolism, providing an accurate snapshot of the microorganism metabolic profile and can be potentially useful for early detection [23,24,25,26,27,28]. Untargeted microbial metabolomic approaches using mass spectrometry or mass spectroscopy-based analytical platforms have become extremely important in the last few years [13,29,30]. Although it is very informative whole metabolome studies can provide a huge volume of data, when it comes to detection of food contamination, only a partial and significant fraction of the metabolic profile is useful and desirable in order to detect the contaminant [13,14]. However, access to properly interpretable data from a specific microbe’s metabolite profile can be a real challenge due to the complexity of these samples. Therefore, the use of high throughput and highly sensitive tools like comprehensive two dimensional gas chromatography (GC×GC) has been used in order to explore and extract useful data from microbial metabolome [22,27,31,32,33,34,35,36,37]. GC×GC provides higher sensitivity and resolution when compared to conventional one-dimensional gas chromatography (^1^D-GC), which fulfils the requirements for the analysis of complex biological samples. Additionally, GC×GC combined with time-of-flight mass spectrometry (ToFMS) increases sensitivity and detectability providing reliable identification of metabolites based on retention rates and mass spectra [27,38,39].

Despite the huge interest in metabolomics patterns in countless processes, application in more complex biological systems of basic metabolomics information obtained from studies carried out in culture media may face several challenges. Several difficulties may occur due to the dynamic nature of the biological systems, and the concentrations of the metabolites may change in response to environmental stimuli. Thus, the aim of this research study was to evaluate the applicability of the *A. niger* volatile metabolite pattern previously established for 3 and 5 days of growth [14], for early contamination detection by direct analysis of table grapes used as study-model. To accomplish this objective, a set of steps was established: (i) 1 day of growth time of *A. niger* cultures; (ii) 1 day of growth time through *A. niger* cultures obtained from previously contaminated grapes; and (iii) direct analysis of contaminated table grapes (in situ assays).

## 2. Materials and Methods

### 2.1. Fungal Strains and Culture Growth Conditions

The three fungal strains used in this study—*Aspergillus niger* (GenBank accession number KT964850), *Penicillium chrysogenum* (GenBank accession number KT799549) and *Candida albicans* (GenBank accession number SC5314)—were obtained from the Department of Biology, University of Aveiro, Portugal. Fresh cultures were prepared by inoculation on Yeast Glucose Chloramphenicol Agar (YGC_A_—20 g L^−1^ D-glucose, 5 g L^−1^ yeast extract, 0.1 g L^−1^ chloramphenicol and 18 g L^−1^ agar; Liofilchem^®^, Roseto degli Abruzzi, Italy).

**Figure 1:**
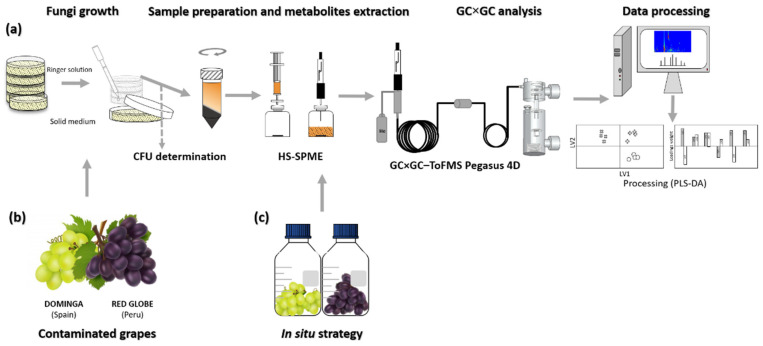
Workflow established for the three strategies used in this study: (**a**) evaluation of the volatile exometabolome for 1 day of growth of *A. niger* cultures, based on [14] (**b**) analysis of *A. niger* cultures obtained from previously contaminated grapes and (**c**) direct analysis of contaminated table grapes with *A. niger* (in situ analysis).

The first experiment was conducted following the same protocol already published for the three fungal strains [14] and adapted in the current study for one day of growth after the inoculation as the workflow presented in Figure 1a. Five plates were prepared with solid YGC_A_ for each assay, where the three fungi were inoculated separately. All essays were performed in triplicate. Each experiment was repeated for 7 days to provide replicates at different times (i.e., three strains with 1-day growth, repeated for 7 days). For each assay, the sampling was performed by adding 10 mL of Ringer solution (Merck Millipore) per plate (5 plates per assay) to collect the cellular content of each sample. After that, 50 mL of the suspension were collected from each assay, an aliquot of 25 mL was collected to volatile metabolites profiling and other aliquot of 25 mL for the determination of cell concentration. The cell concentration was expressed as colony-forming units per milliliter (CFU mL^−1^). The homogenized suspension was serially diluted in Ringer solution and aliquots of 100 μL were spread on YGC_A_ (5 replicates per dilution). These results were employed to normalize the total areas of each chemical feature detected, therefore allowing the determination of specific metabolite production per cell. Finally, to assess general distinction based on metabolomics data, *P. chrysogenum* and *C. albicans* were also plated onto YGC_A_ at 25 °C also performing 5 plates for each assay under study (in a total of 15 plates per condition corresponding to 3 independents assays), and the same procedure used for *A. niger* samples in solid media mentioned above was applied. *Penicillium chrysogenum* was chosen to compare two filamentous fungi from different species, and the unicellular fungus *Candida albicans* was selected for its importance among immunocompromised patients within clinical settings.

### 2.2. Grapes Contamination Protocol

Red globe and Dominga table grapes (*Vitis vinifera* L.) in the commercially mature stage were obtained from a local market in Aveiro, Portugal. The grapes were washed, superficially disinfected with 0.2% (*v*/*v*) sodium hypochlorite for 3 min and rinsed in distilled water to eliminate the residual sodium hypochlorite after being removed from the stems. After drying, the fruits were wounded in a 2 mm depth and 10 μL of an *A. niger* conidial suspension (1 × 10^5^ conidia/mL) was inoculated in the wounded area. Inoculated fruits were kept in a growth chamber under controlled temperature and humidity for seven days until the sporulation stage. Independent assays were performed for red and white varieties.

### 2.3. Profiling Headspace Volatile Metabolites by HS-SPME-GC×GC-ToFMS

#### 2.3.1. Fungal Cultures

The HS-SPME and GC×GC-ToFMS experimental parameters were adapted from a previous study [14], and followed the procedure presented in Figure 1a. After incubation, 25 mL of the suspension mentioned in the previous Section 2.1, was centrifuged at 10,000 rpm, at 4 °C for 15 min (Centrifuge Beckman AVANTI, Indianapolis, IN, USA). Sequentially, 20 mL of supernatant was transferred into a 60 mL glass vial containing 4 g of sodium chloride (NaCl) (≥99.5%, Sigma-Aldrich, St. Louis, MO, USA) and a stirring bar via syringe with 0.20 μm filter. The vials were sealed with a silicone/polytetrafluoroethylene septum and an aluminum cap (Chromacol Ltd., Herts, UK). All the samples were stored at −80 °C until analysis. The SPME extraction was carried out using a 50/30 μm divinylbenzene/carboxen™/polydimethylsiloxane StableFlex™ SPME fibre (DVB/CAR/PDMS). For the HS-SPME protocol, the vials were placed in a thermostatic water bath and headspace extraction was performed for 30 min, at 50 °C, and under continuous agitation at 350 rpm. Three independent aliquots were analyzed for each sample under study, at each day of growth, for seven days.

#### 2.3.2. Instrumentation

SPME fiber was manually introduced into the GC×GC–ToFMS injector and exposed for thermal desorption into heated inlet at 250 °C. The inlet was lined with a 0.75 mm I.D. splitless glass liner and splitless injections mode were used (30 s). The LECO Pegasus 4D (LECO, St. Joseph, MI, USA) GC×GC-ToFMS system was comprised by an Agilent GC 7890A gas chromatograph (Agilent Technologies, Inc., Wilmington, DE, USA), with a dual-stage jet cryogenic modulator (licensed from Zoex) and a secondary oven, as well as mass spectrometer equipped with a ToF analyzer. An Equity-5 column (30 m × 0.32 mm I.D., 0.25 μm film thickness, Supelco, Inc., Bellefonte, PA, USA) and a DB-FFAP column (0.79 m × 0.25 mm I.D., 0.25 μm film thickness, J&W Scientific Inc., Folsom, CA, USA) were used for first (^1^D) and second (^2^D) dimensions, respectively. Helium was employed as carrier gas at a constant flow rate of 2.50 mL min^−1^. The following temperature programs were used: the primary oven temperature was ranged from 40 °C (1 min) to 140 °C at 10 °C min^−1^, and then to 200 °C (1 min) at 7 °C min^−1^. The secondary oven temperature program was 15 °C offset above the primary oven. Both the MS transfer line and MS source temperatures were 250 °C. The modulation period was 5 s, keeping the modulator at 20 °C offset above the primary oven, with hot and cold pulses by periods of 0.80 and 1.70 s, respectively. The ToF analyzer was operated at a spectrum storage rate of 100 spectra s^−1^, with mass spectrometer running in the EI mode at 70 eV and detector voltage of −1480 V, using a *m*/*z* range of 35–300. ChromaTOF^®^ (LECO) GC×GC data processing software was used to process the total ion chromatograms at the signal-to-noise threshold of 200. For identification purposes, the mass spectrum and retention times (^1^D and ^2^D) of the analytes were compared with standards, when available. Additionally, the identification process was done by comparing the mass spectrum of each peak with existing ones in mass spectral libraries, which included an in-house library of standards and two commercial databases (Wiley 275 and US National Institute of Science and Technology (NIST) V. 2.0—Mainlib and Replib). Moreover, additional information such as linear temperature programmed retention indexes (RI) were experimentally determined [40]. For this purpose, the C_8_–C_20_ *n*-alkanes series (the solvent *n*-hexane was used as C_6_ standard) was used for RI determination comparing these values with reported ones in existing literature for chromatographic columns similar to ^1^D column (Table 1). The Deconvoluted Total Ion Current GC×GC area data were used as an approach to estimate the relative content of each metabolite under study.

#### 2.3.3. Contaminated Table Grapes

The contaminated grapes, as described in the Section 2.2, were analyzed using two strategies. The first protocol involved spreading the fungus spores obtained from the contaminated grapes after 7 days of growth into Petri dishes containing YGC_A_ and standardizing the use of two grapes per dish (Figure 1b). After that, the samples were submitted to the same growth conditions and extraction procedures as following the Figure 1a and described before. In this case, five replicates for both varieties of grapes were carried out due to confirm the metabolite profile obtained from them. The second approach employed the in situ HS-SPME methodology to extract directly from the fruits the metabolites produced by the fungus. In this case, ten contaminated grapes were transferred to adapted flasks which enabled them to be hermetically closed, which also included a septum for the SPME fiber introduction. Thus, the in situ experiment was designed to analyze the volatile organic compounds produced by the fungus after 24 h, 4 and 7 days of inoculation. For each time under study, three independent samples were prepared. To avoid cross contamination, for both grape varieties, the inoculation of the fungus was performed in sterilized conditions. In cases where contamination by other species of microorganisms was confirmed, these vials were discarded.

### 2.4. Statistical Analysis

This study consists of two sets of data (Tables S1 and S2). The first data set matrix consists of 54 independent observations, 30 of which refer to 3 fungal species in different growth times (3 and 5 days); 9 referring to a data set in which only the fungus *A. niger* was evaluated in growth times of 1 and 3 days; and 15 addressed to the data set obtained in order to confirm the feasibility of the reduction for the detection time of one day, all replicates referring to *A. niger*. In addition, there are ten independent observations of the profile of metabolites from *A. niger* isolated from grapes (Table S1). The second set of data was obtained from the profile of volatile organic compounds obtained for the same fungus directly in contaminated grapes, using an in situ-SPME approach (Table S2).

All the data set was firstly normalized by colony forming unit per milliliter (CFU mL^−1^) to provide an accurate idea of the metabolite’s concentration per cell. Initially, the set of 44 metabolites detected in a previous study was used to extract information about the new conditions employed (1 day of growth). These metabolites were defined as the *A. niger* molecular biomarkers pattern, and an exploratory test was done to evaluate its potential in fungi distinction. Before the multivariate analysis, the peak areas of the 44 selected analytes were measured for the *A. niger* samples and for the *C. albicans* and *P. chrysogenum* strains. To provide a comprehensive study and a higher number of samples, archival data previously collected in similar experiments in our laboratory were included in the evaluated models, enabling a comparison among all the systems in different growth times and also different periods of experiments (2014, 2016, 2018). The whole data set employed in this study can be found in the Supplementary Materials (Tables S1 and S2). Regarding the multivariate analysis, Principal Component Analysis (PCA) and Partial Least Squares Discriminant Analysis (PLS-DA) strategies were employed using MetaboAnalyst 4.0. The peak areas were previously normalized by the total area, mean-centered and autoscaled, which is a data pre-treatment process that gives variables the same weight. The classification model was statistically validated using leave-one-out cross-validation (LOOCV) which provides an analysis of accuracy, R^2^ (quality-of-fit), and Q^2^ (quality-of-prediction). Model robustness was assessed using a permutation test (1000 permutations).

## 3. Results and Discussion

### 3.1. Evaluating the Potential of A. niger Metabolite Biomarkers Pattern for Strain Distinction at 1-Day of Culture Growth

The initial experiments were performed based on a previous study which included evaluation of the volatile profile of the fungus *A. niger* in comparison with other species and under different growth conditions [14]. Thus, using previously optimized method and other growth conditions, the growth time was reduced to one day in order to minimize the detection time of the fungus in relation to the previous method that spends 3 and 5 days. Table 1 presents the list of the 44 metabolites named as the *A. niger* molecular biomarker pattern [14] and determined by HS-SPME/GC×GC-ToFMS in the samples under study. The 44 metabolites set was chosen since they were identified in all the conditions studied for *A. niger* and, therefore, was defined as a standard of molecular biomarkers for this microorganism. This data set includes hydrocarbons, including aliphatic and aromatic (31.8%), alcohols (22.7%), aldehydes (20.5%), ketones (11.4%), esters (6.8%), terpenic compounds (4.6%), and norisoprenoids (2.3%).

**Figure 2:**
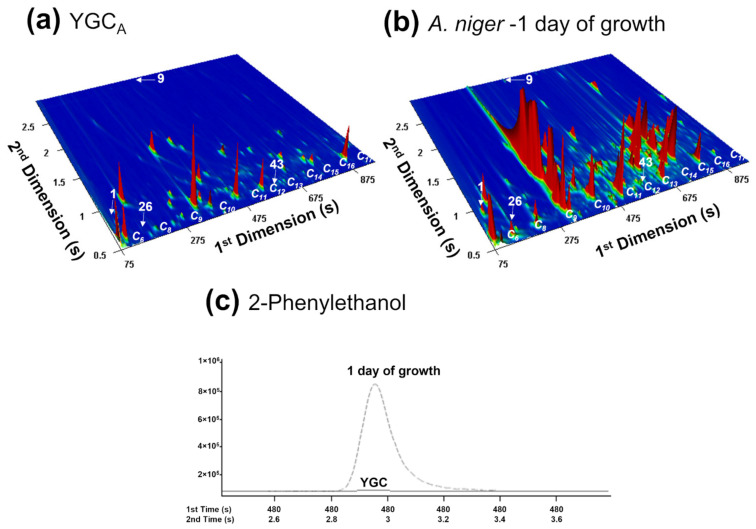
GC × GC-ToFMS total ion chromatogram contour plot of the VOCs released from: (**a**) Yeast Glucose Chloramphenicol Agar medium (YGC_A_), used as control and (**b**) *A. niger* culture headspace volatile components inoculated in YGC_A_, for 1 day of growth, at 37 °C. (**c**) The wide 2-phenylethanol GC × GC peak was identified at a mass spectral acquisition of 100 spectra/s for 1 day of growth and absent for the YGC_A_. Peak number assignment in Table S1 and bold in Table 1.

According to Figure 2, it is possible to verify differences between the culture medium and one day of growth, highlighting the compounds (1) 1-Butanol, (9) 2-Phenylethanol, (26) Toluene, and (43) Endobornyl acetate, assigned in Table S1. For 2-Phenylethanol (Figure 2c), the peak was identified at a mass spectral acquisition of 100 spectra/s for 1 day of growth and absent for the YGC_A_, corroborating the fact that the indicated compounds are directly related to the presence of the microorganism.

**Figure 3:**
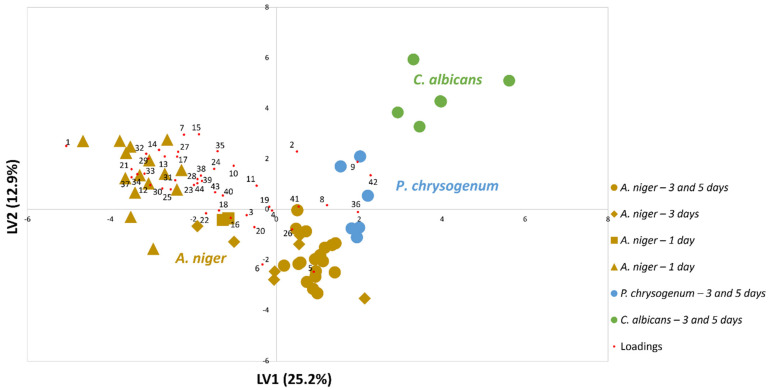
PLS-DA biplot of the samples (LV1×LV2) using the set of 44 metabolites listed in Table 1, which shows a distinction between the three classes of microorganisms under study. The areas were previously standardized by CFU mL^−1^ (Table S1, gray-highlighted). Results obtained by performing the pure culture growth in solid YGC_A_ culture medium and using different growth times.

Therefore, the data set was submitted for evaluation using PLS-DA. This type of tool has been widely used in metabolomics approaches, essentially when each of the selected classes is known. The variation of the samples can be explained by the latent variable which in statistics can be considered the variables that are not directly observed but are rather inferred (through a mathematical model) [53,54]. Figure 3 shows a distinction between the three classes of microorganisms under study. A higher dispersion along LV1 was observed for *A. niger*, which may be explained as this data set comprises samples with 1, 3, and 5 days of growth (Table S1). Additionally, it is possible to observe that most of the related loadings are grouped closed to the *A. niger* samples, which confirms this set of metabolites may be considered as a biomarker pattern of this species. Thus, to verify the classification model established for the data set, it was evaluated using cross-validation.

Cross-validation was performed by using a specific number of samples that are reserved for the construction of the model (training set) and another portion for testing the built model (validation set). The proper choice of cross-validation method from those available basically depends on the number of samples available for use in the training set and validation. In this case, LOOCV was used for the data set in this study once the number of samples is small and it is not possible to assign a sample set to the validation set. The LOOCV consists of choosing one of the samples to compose the validation set and the remaining samples are used for the training set. A new sample is then taken to make up the validation set and the sample that was previously used for the validation set this time will make up the training set; these operations are repeated iteratively several times until all the samples have been part of the validation set at least once [55]. Table 2 presents the results for the statistical parameters obtained in the model validation according to the number of main components used to describe the data.

By looking at the three components, the values of R^2^, Q^2^, and accuracy are 0.9152, 0.8252, and 0.9815, respectively. However, statistical parameters R^2^ and Q^2^ are the most important diagnostic tools for evaluating the performance of the built model. The R^2^ parameter refers to the fitting quality of the model and it is responsible for measuring the performance considering adjusting the raw data. Thus, the values of R^2^ range from 0 to 1, where 1 indicates a perfect model and 0 indicates no adjustment or modeling [56]. The main disadvantage of parameter R^2^ is that it can be adjusted to close to 1 including more terms to the model (in this case, latent variables), and therefore is not a sufficient indicator to assess the validity of the model. Another parameter that should also be evaluated in indicating the utility of a regression model is Q^2^. This parameter is called “predictive quality” and estimates the predictive power of the model, i.e., the model’s ability to predict results from unknown samples. Q^2^ represents a more realistic and useful performance indicator since it reflects the final objective of modeling that refers to predictions for new experiments. Thus, R^2^ and Q^2^ have an upper limit of 1 and the validity of a model is related to the high values of R^2^ and Q^2^. In general, Q^2^ > 0.5 can be considered good and Q^2^ > 0.9 excellent. Accuracy, in general, is used to describe the proximity of a measure to a value taken as true. In classification models, accuracy represents the number of correctly predicted samples in relation to the total of samples in the test set. Always use as few components as possible in the construction of the model from which there is no significant gain in the accuracy and other parameters of the model evaluation, and this should be done by keeping the values of these parameters close to 1, thus ensuring the high predictability of the model. The use of more components than necessary results in the inclusion of unnecessary information to the model, making it over-adjusted and thus compromising its predictive ability. Thus, taking into account that the construction of a model based on living organisms and its metabolic production may vary according to environmental conditions and other external factors, the values obtained by the parameters R^2^, Q^2^ and accuracy ensure the rationality of the model built for this study, making it possible to use in the prediction of new samples [55,57].

Compounds such as 2-phenylethanol, 3-octanol, 3-methyl-1-butanol are among the most important variables and have already been described in the literature as characteristic volatile metabolites of fungal species, including *A. niger* [13,14,58]. Volatile organic compounds such as geosmin, methylisoborneol, 1-octen-3-ol, 1-octen-3-one and 3-octanone have been reported as common to several fungal species and also, sensory differences between wines made with grapes contaminated with different kinds of bunch rots have already been described [13]. In this sense, it is important to choose compounds that can be used as unique biomarkers for classes of microorganisms, as this targeted approach facilitates their identification without the use of highly complex data. Thus, statistical models can be concluded based on these metabolites to evaluate if this selection is sufficient and valid for a classification model.

### 3.2. Following the A. niger Metabolites Biomarkers Pattern over 7 Days

#### 3.2.1. By Analysis of *A. niger* Cultures Obtained from Contaminated Grapes

**Figure 4:**
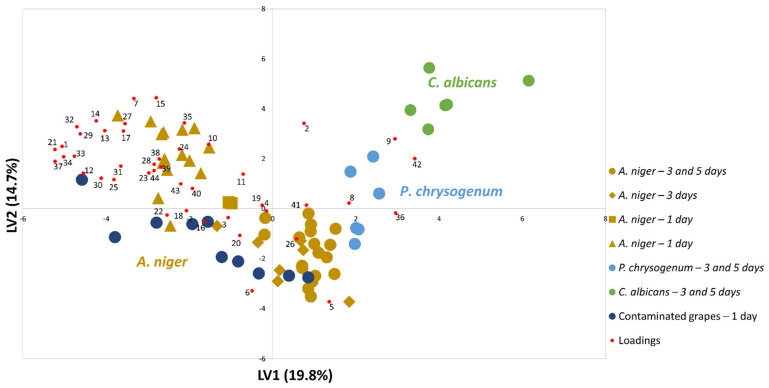
PLS-DA biplot of samples (LV1×LV2) using the set of 44 metabolites listed in Table 1, which shows that the grapes contaminated with *A. niger* presents a metabolomics pattern like the pure cultures. The areas were previously standardized by CFU mL^−1^ (Table S1). Results obtained by performing the pure culture growth in solid YGC_A_ culture medium and by contaminated grapes (Red Globe and Dominga) with *A. niger*.

Based on the previous model, an application study was carried out to detect *A. niger* in contaminated grapes. The same growth strategies, analysis, and data treatment were employed, and the data obtained were tested to predict the contamination of the samples with this pathogen. Thus, after 7 days of growth, the spores of *A. niger* in the grapes were transferred to petri dishes with YGC_A_ medium. For this procedure, two grapes per plate were used and the experiments were performed in five repetitions for each grape variety. Figure 4 presents the complete data set, using the same previous model, including the samples from contaminated grapes.

According to Figure 4, the set of data obtained with the contaminated grapes is similar to the previous data obtained to construct the model, following the PLS-DA graph. Therefore, the fungus can be detected after only one day of infection. The interest in detecting the presence of microorganisms has been widely studied [59,60], with applications employed in the hospital area [61,62], the environment [63], and mainly food [12,64], including the fungus *A. niger* [65]. Nevertheless, the differential of this study lies in the fact that the detection of contamination by *A. niger* can be performed in only one day involving all the steps described above, which is quite significant when compared to traditional methods dependent on the isolation and characterization of infectious agents; usually a very long and troublesome path. Although the protocol is not an identification that can completely replace the use of molecular tools, the strategy employed can assist in the early investigation due to detection or not of the microorganisms’ presence before using other approaches for accurate identification.

#### 3.2.2. By Direct Analysis of Contaminated Table Grapes—In Situ SPME

**Figure 5:**
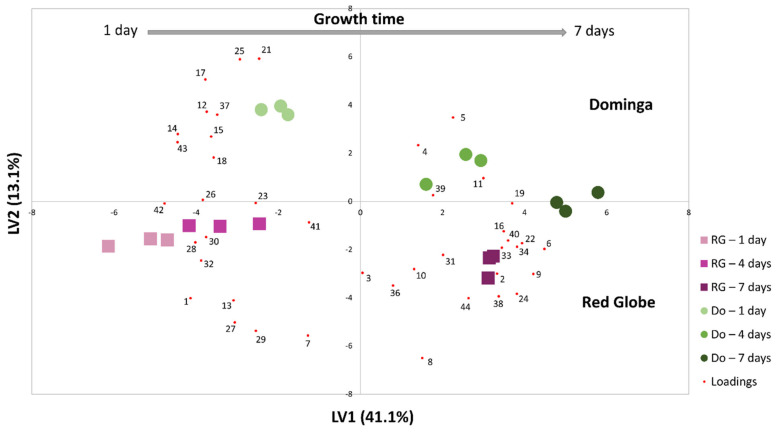
PLS-DA biplot of samples (LV1×LV2) using the set of 42 metabolites listed in Table 2, showing clear distinction between both contaminated grape varieties over time. Results obtained over 7 days (Table S2) for White (Dominga—Do) and Red (Red Globe—RG) grapes contaminated with *A. niger*. Independent assays were performed for red and white varieties.

The followed volatile metabolite profile of the disease was evaluated through 7 days of growth by using 42 metabolites associated with *A. niger* metabolism, because in this study only 42 of the total set of metabolites were identified in grapes. By employing this strategy, 2-methyl-6-phenyl-1,6-heptadiene and methyl 2-methylpropenoate were not found. Although there is no clear indication regarding their absence, the differences in the substrate can slightly modify metabolite production, indicating the importance of in situ studies. An in situ SPME strategy was performed using a non-invasive headspace approach. Data were equally subjected to statistical analysis using MetaboAnalyst 4.0 tool to identify possible groupings of the investigated samples. Figure 5 shows that there was a clear separation between the samples, associated with a variation in the concentration of metabolites over the days of fungal growth. In vivo SPME studies have been widely applied in food analysis, since coated surfaces are biocompatible and also provide higher sensitivity and reduced analysis time [66,67,68].

PLS-DA scores plot (Figure 5) revealed the dispersion of the samples according to main factors; time of growth and variety. The latent variable 1 (LV1) is mainly responsible for explaining the data variability (41.1%), which showed dispersion of the samples according to the growth time, from 1 day (LV1 negative) to 7 days (LV1 positive). Along LV2, which explained 13.1% of the data variability, the samples are organized according to the variety, with Do mainly in the LV2 positive and the RG in the LV2 negative.

In addition, from these data it was also possible to infer that red variety (RG) was more resistant to contamination than white one (Do), as for RG after 4 days of contamination the metabolite profile was still quite similar to that of the first day. This fact can be explained by the higher concentration of phenolic and antioxidant compounds in general present in this type of grape, ensuring a higher resistance to contamination. A recent study demonstrated the antagonistic activity of essential oils of eight plants against strains of *A. niger*, and one of the factors highlighted by the authors was the presence of antioxidant compounds that helped to control the disease in stored wheat grains [69,70]. Fu-xiang et al. demonstrated that fresh grapes are rich in bioactive phenolic compounds, and the concentration depends heavily on the grape varieties. The Mascot Kyoho variety (red skin) had the highest concentration of antioxidant compounds such as (+)-catechin, (−)-epicatechin, rutin, isoquercitrin, and kaempferol. The phenolic compounds of grapes are mainly in their skins, and this corroborates the results shown above, where the high content of antioxidant compounds promotes resistance to the growth of the pathogen. The effect of inhibiting the growth of *A. niger* against antioxidant enzymes has been previously studied and possibly suggests the induction of antioxidant defense response by Trichoderma bio- controller to combat oxidative bursts produced by invading pathogens [70,71].

Additionally, the dendrogram and heatmap (Figure S1) illustrate the formation of two main clusters related to the two grape varieties, within each one there is organization of the samples as a function of growth time. The biplot (Figure 5) and heatmaps (Figure S1) demonstrate that the most important compounds directly related to *A. niger* contamination are 3-octanol, 2-phenylethanol, 3-methyl-butanol, and 1-octen-3ol (only for Dominga). Based on previously reported studies, all of these compounds can be associated to volatile organic compounds produced by microorganism species [15,27,72], even *A. niger* [13,14]. Previous studies have demonstrated the pathway for the production of 1-octanol is from glucose and fatty acids in *E. coli* and this compound is mainly accumulated in the culture media fraction [15]. Furthermore, 2-phenyletanol and 3-methyl-butanol compounds are very common as microorganism biomarkers. Production of these metabolites are associated with the isoleucine/leucine and phenylalanine pathway metabolism, respectively [27,72,73].

Non-invasive methods such as HS-SPME are very useful in the detection and monitoring of food contamination, including the level of contamination. Recent works have demonstrated the utilization of metabolomic strategies in the detection of several microorganisms in grapes and grape juices [4,13,14]. In general, the approaches utilized involve a non-target metabolic exploration step in order to verify a pattern of compounds responsible for the contamination and from this point on, only these target compounds are monitored to verify different levels of contamination [13,74]. This was also the approach initially used during this work, and therefore justifies the choice of the set of metabolites investigated.

The data obtained so far demonstrate that it is possible to detect contamination by *A. niger* directly in grapes using a non-invasive and rapid method. From this initial information, new statistical tools and approaches will be employed in order to explore these data in more depth and obtain important information regarding the profile of volatile metabolites and their relationship with the level of fungal contamination [69,75].

## 4. Conclusions

In conclusion, the developed tools can be used to ensure the quality and food safety of grapes by employing early and sophisticated metabolomics strategies. It was possible to perform the construction of an experimental model from previously selected metabolites by reducing the growth time to one day. The model was validated employing statistical tests, and the results were considered satisfactory in modeling the microorganism’s behavior. In addition, the model was tested to verify the presence of the microorganism *A. niger* in contaminated grapes for two varieties, and proved the presence of the microorganism even with one day of detection time. Fungus detection from the set of metabolites was also used for kinetic follow-up of fungus growth over 7 days. Using a PLS-DA approach, it was possible to observe that the matrix is an important factor for fungi development, as different concentrations of the metabolites were observed for the red and white grape varieties. The results obtained throughout this study suggest that even when the analysis was performed for the samples with one day of growth time, the pattern of metabolites associated with the *A. niger* species was detected, which may be extremely useful in making this type of method possible for the rapid detection of food contamination.

Moreover, it is hoped that the methodology developed can be extended to other problems involving the detection of this microorganism, such as its detection in other foods, or even in other environments where it may be present in a way that is harmful to human health. Although this article represents a step forward in the evaluation of the applicability of the metabolome of *A. niger* in contaminated real samples, for its further application in other scenarios, it is mandatory to look for other parameters, such as limits of detection, evaluation of confounders, matrix effects, and the presence of a mixture of species (co-cultures), among others.

## Figures and Tables

**Table 1:** List of the 44 metabolites named as the *A. niger* molecular biomarker pattern and determined by HS-SPME/GC×GC-ToFMS.

Peak Number	^1^*t*_R_^a^ (s)	^2^*t*_R_^a^ (s)	Metabolite	CAS Number	Formula	MSI Level ^b^	RI_Calc_^c^	RI_Lit_^d^
1	115	0.910	1-Butanol	71-36-3	C_4_H_10_O	1	644	655 [41]
2	150	1.160	3-Methyl-1-butanol	123-51-3	C_5_H_12_O	1	718	706 [42]
3	255	1.140	1-Hexanol	111-27-3	C_6_H_14_O	1	878	877 [42]
4	345	1.100	1-Heptanol	111-70-6	C_7_H_16_O	2	975	974 [43]
5	350	1.050	1-Octen-3-ol	3391-86-4	C_8_H_16_O	1	980	992 [44]
6	365	1.270	3-Octanol	589-98-0	C_8_H_18_O	1	996	996 [45]
7	395	0.990	2-Ethyl-1-hexanol	104-76-7	C_8_H_18_O	2	1029	1038 [44]
8	440	1.030	1-Octanol	111-87-5	C_9_H_18_O_2_	1	1079	1079 [44]
9	475	3.030	2-Phenylethanol	60-12-8	C_8_H_10_O	1	1120	1107 [46]
10	805	2.060	2,4-bis(1,1-Dimethylethyl)phenol	96-76-4	C_14_H_22_O	2	1514	1513 [47]
11	110	0.460	3-Methylbutanal	590-86-3	C_5_H_10_O	2	633	628 [48]
12	190	0.590	Hexanal	66-25-1	C_6_H_12_O	1	801	800 [49]
13	275	0.620	Heptanal	111-71-7	C_7_H_14_O	1	901	903 [49]
14	465	0.630	Nonanal	124-19-6	C_9_H_18_O	1	1106	1106 [42]
15	555	0.630	Decanal	112-31-2	C_10_H_20_O	2	1207	1206 [49]
16	685	0.770	2-Undecenal	2463-77-6	C_11_H_20_O	2	1364	1369 [50]
17	720	0.650	Dodecanal	112-54-9	C_12_H_24_O	2	1407	1406 [49]
18	335	1.550	Benzaldehyde	100-52-7	C_7_H_6_O	1	965	954 [51]
19	410	1.620	Benzeneacetaldehyde	122-78-1	C_8_H_8_O	1	1046	1049 [41]
20	135	0.530	Methyl 2-methylpropenoate	80-62-6	C_5_H_8_O_2_	2	685	710 [41]
21	695	0.920	3-Hydroxy-2,4,4-trimethylpentyl 2-methylpropanoate	74367-34-3	C_12_H_24_O_3_	2	1376	1376 [13]
22	545	1.050	2-Phenylethylacetate	103-45-7	C_10_H_12_O_2_	2	1196	1196 [13]
23	880	0.490	Hexadecane	544-76-3	C_16_H_34_	1	1601	1600 [49]
24	965	0.430	Heptadecane	629-78-7	C_17_H_36_	1	1701	1700 [49]
25	115	0.460	Benzene	71-43-2	C_6_H_6_	1	643	643 [13]
26	170	0.540	Toluene	108-88-3	C_7_H_8_	1	759	771 [41]
27	250	0.590	1,3-Dimethylbenzene	108-38-3	C_8_H_10_	2	871	871 [13]
28	270	0.640	1,2-Dimethylbenzene	95-47-6	C_8_H_10_	2	901	900 [42]
29	325	0.580	Propylbenzene	103-65-1	C_9_H_12_	2	953	959 [41]
30	335	0.590	1-Ethyl-4-methylbenzene	622-96-8	C_9_H_12_	2	964	970 [41]
31	365	0.640	1,3,5-Trimethylbenzene	108-67-8	C_9_H_12_	2	995	974 [41]
32	390	0.580	1-Methyl-2-(1-methylethyl)benzene	527-84-4	C_10_H_14_	2	1023	1023 [13]
33	390	0.690	1,2,3-Trimethylbenzene	526-73-8	C_9_H_12_	1	1023	1023 [13]
34	425	0.610	2-Ethyl-1,4-dimethylbenzene	1758-88-9	C_10_H_14_	2	1062	1062 [13]
35	700	1.270	Biphenyl	92-52-4	C_12_H_10_	2	1383	1383 [13]
36	880	1.020	2-Methyl-6-phenyl-1,6-heptadiene	51708-97-5	C_14_H_18_	2	1601	1601 [13]
37	75	0.390	2-Propanone	67-64-1	C_3_H_6_O	1	559	559 [13]
38	265	0.580	3-Heptanone	106-35-4	C_7_H_14_O	1	889	884 [41]
39	355	0.740	6-Methyl-5-hepten-2-one	110-93-0	C_8_H_14_O	1	985	985 [41]
40	495	0.760	3-Nonen-2-one	18402-83-0	C_9_H_16_O	2	1140	1140 [13]
41	755	0.800	6,10-Dimethyl-5,9-undecadien-2-one	3796-70-1	C_13_H_22_O	2	1451	1451 [13]
42	435	0.790	2,6-Dimethyl-7-octen-2-ol	18479-58-8	C_10_H_20_O	2	1073	1073 [13]
43	625	0.630	Endobornyl acetate	76-49-3	C_12_H_20_O_2_	2	1289	1289 [13]
44	780	0.750	α-Methylionone	127-51-5	C_14_H_22_O	2	1482	1482 [13]

^a^ Retention times for first (^1^ *t*_R_) and second (^2^ *t*_R_) dimensions in seconds. ^b^ Level of metabolite identification according to Sumner et al. [52]. (1) Identified compounds; (2) Putatively annotated compounds; (3) Putatively characterized compound classes; (4) Unknown compounds. ^c^ RI_Calc_: Linear Retention Index obtained through the modulated chromatogram. ^d^ RI_Lit_: Linear Retention Index reported in the literature for Equity-5 column or equivalents.

**Table 2:** Statistical parameters for the evaluation of the PLS-DA model according to the number of components for the classification model.

Parameters	Latent Variable 1	Latent Variable 2	Latent Variable 3	Latent Variable 4	Latent Variable 5
Accuracy	0.7778	0.7778	0.9815	1.0	1.0
R^2^	0.4548	0.8821	0.9152	0.9312	0.9557
Q^2^	0.2957	0.7888	0.8252	0.81084	0.8128

## Supplementary Materials

The following are available online at https://www.mdpi.com/article/10.3390/foods10112870/s1, Figure S1: Dendrogram and heatmap representation of the 42 metabolites putatively identified from red (RG) and white (Do) table grapes contaminated with *A. niger*, determined by in situ HS-SPME/GC×GC-ToFMS over 7 days, which illustrates the formation of two main clusters related to the two grape varieties, within each one there is an organization of the samples as a function of the growth time. Table S1. Full data set used for statistical processing including the chromatographic data of the 44 metabolites putatively identified and normalized by CFU mL^−1^ from *A. niger*, *P. chrysogenum* and *C. albicans* cultures in different growth times, using HS-SPME/GC×GC-ToFMS. Results obtained by performing the pure culture growth in solid YGC_A_ culture medium and by contaminated grapes (Red Globe and Dominga) with *A. niger* after 7 days of growth. Table S2. Full-data set used for statistical processing including the chromatographic data of the 42 metabolites putatively identified from red and white tables grapes contaminated with *A. niger* and determined using in situ-HS-SPME/GC×GC-ToFMS.

## References

[1] Finger J.A.F.F., Baroni W.S.G.V., Maffei D.F., Bastos D.H.M., Pinto U.M. (2019). Overview of foodborne disease outbreaks in Brazil from 2000 to 2018. Foods.

[2] Gómez J.V., Tarazona A., Mateo F., Jiménez M., Mateo E.M. (2019). Potential impact of engineered silver nanoparticles in the control of aflatoxins, ochratoxin A and the main aflatoxigenic and ochratoxigenic species affecting foods. Food Control.

[3] Sun Q., Li J., Sun Y., Chen Q., Zhang L., Le T. (2020). The antifungal effects of cinnamaldehyde against *Aspergillus niger* and its application in bread preservation. Food Chem..

[4] Bazioli J.M., Belinato J.R., Costa J.H., Akiyama D.Y., Pontes J.G., Kupper K.C., Augusto F., Fill T.P. (2019). Biological Control of Citrus Postharvest Phytopathogens. Toxins.

[5] Freire L., Guerreiro T.M., Pia A.K.R., Lima E.O., Oliveira D.N., Melo C.F.O.R., Catharino R.R., Sant’Ana A.S. (2018). A quantitative study on growth variability and production of ochratoxin A and its derivatives by *A. carbonarius* and *A. niger* in grape-based medium. Sci. Rep..

[6] Li Q., Li C., Li P., Zhang H., Zhang X., Zheng X., Yang Q., Apaliya M.T., Boateng N.A.S., Sun Y. (2017). The biocontrol effect of Sporidiobolus pararoseus Y16 against postharvest diseases in table grapes caused by *Aspergillus niger* and the possible mechanisms involved. Biol. Control.

[7] Freire L., Braga P.A.C., Furtado M.M., Delafiori J., Dias-Audibert F.L., Pereira G.E., Reyes F.G., Catharino R.R., Sant’Ana A.S. (2020). From grape to wine: Fate of ochratoxin A during red, rose, and white winemaking process and the presence of ochratoxin derivatives in the final products. Food Control.

[8] Cano A., Cháfer M., Chiralt A., González-Martínez C. (2015). Physical and Antimicrobial Properties of Starch-PVA Blend Films as Affected by the Incorporation of Natural Antimicrobial Agents. Foods.

[9] Abarca M.L., Bragulat M.R., Castellá G., Cabañes F.J. (2019). Impact of some environmental factors on growth and ochratoxin A production by *Aspergillus niger* and *Aspergillus welwitschiae*. Int. J. Food Microbiol..

[10] dos Santos-Ciscon B.A., van Diepeningen A., da Cruz Machado J., Dias I.E., Waalwijk C. (2019). *Aspergillus* species from Brazilian dry beans and their toxigenic potential. Int. J. Food Microbiol..

[11] Dachery B., Hernandes K.C., Veras F.F., Schmidt L., Augusti P.R., Manfroi V., Zini C.A., Welke J.E. (2019). Effect of *Aspergillus carbonarius* on ochratoxin a levels, volatile profile and antioxidant activity of the grapes and respective wines. Food Res. Int..

[12] Gil-Serna J., García-Díaz M., Vázquez C., González-Jaén M.T., Patiño B. (2019). Significance of *Aspergillus niger* aggregate species as contaminants of food products in Spain regarding their occurrence and their ability to produce mycotoxins. Food Microbiol..

[13] Schueuermann C., Steel C.C., Blackman J.W., Clark A.C., Schwarz L.J., Moraga J., Collado I.G., Schmidtke L.M. (2019). A GC–MS untargeted metabolomics approach for the classification of chemical differences in grape juices based on fungal pathogen. Food Chem..

[14] Costa C.P., Gonçalves Silva D., Rudnitskaya A., Almeida A., Rocha S.M. (2016). Shedding light on *Aspergillus niger* volatile exometabolome. Sci. Rep..

[15] Akhtar M.K., Dandapani H., Thiel K., Jones P.R. (2015). Microbial production of 1-octanol: A naturally excreted biofuel with diesel-like properties. Metab. Eng. Commun..

[16] Erban A., Fehrle I., Martinez-Seidel F., Brigante F., Más A.L., Baroni V., Wunderlin D., Kopka J. (2019). Discovery of food identity markers by metabolomics and machine learning technology. Sci. Rep..

[17] Zhang X.W., Li Q.H., Xu Z.D., Dou J.J. (2020). Mass spectrometry-based metabolomics in health and medical science: A systematic review. RSC Adv..

[18] Wang L., Meeus I., Rombouts C., Van Meulebroek L., Vanhaecke L., Smagghe G. (2019). Metabolomics-based biomarker discovery for bee health monitoring: A proof of concept study concerning nutritional stress in *Bombus terrestris*. Sci. Rep..

[19] Xanthopoulou A., Ganopoulos I., Tryfinopoulou P., Panagou E.Z., Osanthanunkul M., Madesis P., Kizis D. (2019). Rapid and accurate identification of black aspergilli from grapes using high-resolution melting (HRM) analysis. J. Sci. Food Agric..

[20] Järvinen A.K., Laakso S., Piiparinen P., Aittakorpi A., Lindfors M., Huopaniemi L., Piiparinen H., Mäki M. (2009). Rapid identification of bacterial pathogens using a PCR- and microarray-based assay. BMC Microbiol..

[21] Schloter M., Abmus B., Hartmann A. (1995). The Use of Immunological Methods To Detect and Identify Bacteria in the Environment. Biotech. Adv..

[22] Fang S., Liu S., Song J., Huang Q., Xiang Z. (2021). Recognition of pathogens in food matrixes based on the untargeted *in vivo* microbial metabolite profiling via a novel SPME/GC × GC-QTOFMS approach. Food Res. Int..

[23] Baptista I., Santos M., Rudnitskaya A., Saraiva J.A., Almeida A. (2019). A comprehensive look into the volatile exometabolome of enteroxic and non-enterotoxic *Staphylococcus aureus* strains. Int. J. Biochem. Cell Biol..

[24] Martins C., Brandão T., Almeida A., Rocha S.M. (2017). Metabolomics strategy for the mapping of volatile exometabolome from *Saccharomyces* spp. widely used in the food industry based on comprehensive two-dimensional gas chromatography. J. Sep. Sci..

[25] Fialho M.B., Toffano L., Pedroso M.P., Augusto F., Pascholati S.F. (2010). Volatile organic compounds produced by *Saccharomyces cerevisiae* inhibit the in vitro development of *Guignardia citricarpa*, the causal agent of citrus black spot. World J. Microbiol. Biotechnol..

[26] Belinato J.R., Dias F.F.G., Caliman J.D., Augusto F., Hantao L.W. (2018). Opportunities for green microextractions in comprehensive two-dimensional gas chromatography / mass spectrometry-based metabolomics—A review. Anal. Chim. Acta.

[27] De Souza J.R.B., Kupper K.C., Augusto F. (2018). In vivo investigation of the volatile metabolome of antiphytopathogenic yeast strains active against *Penicillium digitatum* using comprehensive two-dimensional gas chromatography and multivariate data analysis. Microchem. J..

[28] Zeiss D.R., Mhlongo M.I., Tugizimana F., Steenkamp P.A., Dubery I.A. (2019). Metabolomic profiling of the host response of tomato (*Solanum lycopersicum*) following infection by *Ralstonia solanacearum*. Int. J. Mol. Sci..

[29] Cubero-Leon E., Peñalver R., Maquet A. (2014). Review on metabolomics for food authentication. Food Res. Int..

[30] Wang L., Qu L., Zhang L., Hu J., Tang F., Lu M. (2016). Metabolic responses of poplar to *Apripona germari* (Hope) as revealed by metabolite profiling. Int. J. Mol. Sci..

[31] Alves Z., Melo A., Figueiredo A.R., Coimbra M.A., Gomes A.C., Rocha S.M. (2015). Exploring the *saccharomyces cerevisiae* volatile metabolome: Indigenous versus commercial strains. PLoS ONE.

[32] Cardoso P., Santos M., Freitas R., Rocha S.M., Figueira E. (2017). Response of *Rhizobium* to Cd exposure: A volatile perspective. Environ. Pollut..

[33] Fonseca A.M.A., Dias C., Amaro A.L., Isidoro N., Pintado M., Silvestre A.J.D., Rocha S.M. (2020). The impact of plant-based coatings in “ROCHA” pear preservation during cold storage: A metabolomic approach. Foods.

[34] Martins C., Brandão T., Almeida A., Rocha S.M. (2020). Enlarging knowledge on lager beer volatile metabolites using multidimensional gas chromatography. Foods.

[35] Matos D., Sá C., Cardoso P., Pires A., Rocha S.M., Figueira E. (2019). The role of volatiles in *Rhizobium* tolerance to cadmium: Effects of aldehydes and alcohols on growth and biochemical endpoints. Ecotoxicol. Environ. Saf..

[36] Mousavi F., Bojko B., Bessonneau V., Pawliszyn J. (2016). Cinnamaldehyde Characterization as an Antibacterial Agent toward *E. coli* Metabolic Profile Using 96-Blade Solid-Phase Microextraction Coupled to Liquid Chromatography-Mass Spectrometry. J. Proteome Res..

[37] Rees C.A., Franchina F.A., Nordick K.V., Kim P.J., Hill J.E. (2017). Expanding the *Klebsiella pneumoniae* volatile metabolome using advanced analytical instrumentation for the detection of novel metabolites. J. Appl. Microbiol..

[38] Parastar H., Garreta-Lara E., Campos B., Barata C., Lacorte S., Tauler R. (2018). Chemometrics comparison of gas chromatography with mass spectrometry and comprehensive two-dimensional gas chromatography with time-of-flight mass spectrometry *Daphnia magna* metabolic profiles exposed to salinity. J. Sep. Sci..

[39] Carriço Í.R., Marques J., Trujillo-Rodriguez M.J., Anderson J.L., Rocha S.M. (2020). Sorbent coatings for solid-phase microextraction targeted towards the analysis of death-related polar analytes coupled to comprehensive two-dimensional gas chromatography: Comparison of zwitterionic polymeric ionic liquids versus commercial coatings. Microchem. J..

[40] Van Den Dool H., Kratz P.D. (1963). A generalization of the retention index system including linear temperature programmed gas-liquid partition chromatography. J. Chromatogr. A.

[41] Xu X., Van Stee L.L.P., Williams J., Beens J., Adahchour M., Vreuls R.J.J., Brinkman U.A.T., Lelieveld J. (2003). Comprehensive two-dimensional gas chromatography (GC×GC) measurements of volatile organic compounds in the atmosphere. Atmos. Chem. Phys..

[42] Rocha S.M., Freitas R., Cardoso P., Santos M., Martins R., Figueira E. (2013). Exploring the potentialities of comprehensive two-dimensional gas chromatography coupled to time of flight mass spectrometry to distinguish bivalve species: Comparison of two clam species (*Venerupis decussata* and *Venerupis philippinarum*). J. Chromatogr. A.

[43] Salvador Â.C., Baptista I., Barros A.S., Gomes N.C.M., Cunha Â., Almeida A., Rocha S.M. (2013). Can Volatile Organic Metabolites Be Used to Simultaneously Assess Microbial and Mite Contamination Level in Cereal Grains and Coffee Beans?. PLoS ONE.

[44] Silva I., Rocha S.M., Coimbra M.A., Marriott P.J. (2010). Headspace solid-phase microextraction combined with comprehensive two-dimensional gas chromatography time-of-flight mass spectrometry for the determination of volatile compounds from marine salt. J. Chromatogr. A.

[45] Oliveira D.R., Leitão G.G., Santos S.S., Bizzo H.R., Lopes D., Alviano C.S., Alviano D.S., Leitão S.G. (2006). Ethnopharmacological study of two *Lippia* species from Oriximiná, Brazil. J. Ethnopharmacol..

[46] Weldegergis B.T., Crouch A.M., Górecki T., de Villiers A. (2011). Solid phase extraction in combination with comprehensive two-dimensional gas chromatography coupled to time-of-flight mass spectrometry for the detailed investigation of volatiles in South African red wines. Anal. Chim. Acta.

[47] Zhao C., Li X., Liang Y., Fang H., Huang L.-F., Guo F. (2006). Comparative analysis of chemical components of essential oils from different samples of Rhododendron with the help of chemometrics methods. Chemom. Intell. Lab. Syst..

[48] Loureiro C.C., Duarte I.F., Gomes J., Carrola J., Barros A.S., Gil A.M., Bousquet J., Bom A.T., Rocha S.M. (2014). Urinary metabolomic changes as a predictive biomarker of asthma exacerbation. J. Allergy Clin. Immunol..

[49] Rocha S.M., Caldeira M., Carrola J., Santos M., Cruz N., Duarte I.F. (2012). Exploring the human urine metabolomic potentialities by comprehensive two-dimensional gas chromatography coupled to time of flight mass spectrometry. J. Chromatogr. A.

[50] Liu Y., Xu X.L., Zhou G.H. (2007). Comparative study of volatile compounds in traditional Chinese Nanjing marinated duck by different extraction techniques. Int. J. Food Sci. Technol..

[51] Caldeira M., Barros A.S., Bilelo M.J., Parada A., Câmara J.S., Rocha S.M. (2011). Profiling allergic asthma volatile metabolic patterns using a headspace-solid phase microextraction/gas chromatography based methodology. J. Chromatogr. A.

[52] Sumner L.W., Amberg A., Barrett D., Beale M.H., Beger R., Daykin C.A., Fan T.W.M., Fiehn O., Goodacre R., Griffin J.L. (2007). Proposed minimum reporting standards for chemical analysis: Chemical Analysis Working Group (CAWG) Metabolomics Standards Initiative (MSI). Metabolomics.

[53] Adamiak J., Bonifay V., Otlewska A., Sunner J.A., Beech I.B., Stryszewska T., Kanka S., Oracz J., Zyzelewicz D., Gutarowska B. (2017). Untargeted metabolomics approach in halophiles: Understanding the biodeterioration process of building materials. Front. Microbiol..

[54] Pan X., Liu H., Liu J., Wang C., Wen J. (2016). Omics-based approaches reveal phospholipids remodeling of *Rhizopus oryzae* responding to furfural stress for fumaric acid-production from xylose. Bioresour. Technol..

[55] Triba M.N., Le Moyec L., Amathieu R., Goossens C., Bouchemal N., Nahon P., Rutledge D.N., Savarin P. (2015). PLS/OPLS models in metabolomics: The impact of permutation of dataset rows on the K-fold cross-validation quality parameters. Mol. Biosyst..

[56] Li S., Hu Y., Liu W., Chen Y., Wang F., Lu X., Zheng W. (2020). Untargeted volatile metabolomics using comprehensive two-dimensional gas chromatography-mass spectrometry—A solution for orange juice authentication. Talanta.

[57] Fan S., Shahid M., Jin P., Asher A., Kim J. (2020). Identification of Metabolic Alterations in Breast Cancer Using Mass Spectrometry-Based Metabolomic Analysis. Metabolites.

[58] Zhao G., Yin G., Inamdar A.A., Luo J., Zhang N., Yang I., Buckley B., Bennett J.W. (2017). Volatile organic compounds emitted by filamentous fungi isolated from flooded homes after Hurricane Sandy show toxicity in a Drosophila bioassay. Indoor Air.

[59] Rees C.A., Burklund A., Stefanuto P.H., Schwartzman J.D., Hill J.E. (2018). Comprehensive volatile metabolic fingerprinting of bacterial and fungal pathogen groups. J. Breath Res..

[60] Sarrocco S., Vannacci G. (2018). Preharvest application of beneficial fungi as a strategy to prevent postharvest mycotoxin contamination: A review. Crop. Prot..

[61] Pantoja L.D.M., do Nascimento R.F., de Araujo Nunes A.B. (2016). Investigation of fungal volatile organic compounds in hospital air. Atmos. Pollut. Res..

[62] Tong H., Wang Y., Li Y., Liu S., Chi C., Liu D., Guo L., Li E., Wang C. (2017). Volatile organic metabolites identify patients with gastric carcinoma, gastric ulcer, or gastritis and control patients. Cancer Cell Int..

[63] Aquino S., de Lima J.E.A., do Nascimento A.P.B., Reis F.C. (2018). Analysis of fungal contamination in vehicle air filters and their impact as a bioaccumulator on indoor air quality. Air Qual. Atmos. Heal..

[64] Cumeras R., Aksenov A.A., Pasamontes A., Fung A.G., Cianchetta A.N., Doan H., Davis R.M., Davis C.E. (2016). Identification of fungal metabolites from inside *Gallus gallus domesticus* eggshells by non-invasively detecting volatile organic compounds (VOCs). Anal. Bioanal. Chem..

[65] Jedidi I., Cruz A., González-Jaén M.T., Said S. (2017). Aflatoxins and ochratoxin A and their *Aspergillus* causal species in Tunisian cereals. Food Addit. Contam. Part B Surveill..

[66] Zhang Q.H., Zhou L.D., Chen H., Wang C.Z., Xia Z.N., Yuan C.S. (2016). Solid-phase microextraction technology for in vitro and in vivo metabolite analysis. TrAC Trends Anal. Chem..

[67] Souza-Silva É.A., Reyes-Garcés N., Gómez-Ríos G.A., Boyaci E., Bojko B., Pawliszyn J. (2015). A critical review of the state of the art of solid-phase microextraction of complex matrices III. Bioanalytical and clinical applications. TrAC Trends Anal. Chem..

[68] Risticevic S., Souza-Silva E.A., Gionfriddo E., DeEll J.R., Cochran J., Hopkins W.S., Pawliszyn J. (2020). Application of *in vivo* solid phase microextraction (SPME) in capturing metabolome of apple (*Malus × domestica Borkh*) fruit. Sci. Rep..

[69] Kumar P., Mishra S., Kumar A., Kumar S., Prasad C.S. (2017). In vivo and in vitro control activity of plant essential oils against three strains of *Aspergillus niger*. Environ. Sci. Pollut. Res..

[70] Li F.-x., Li F.-h., Yang Y.-x., Yin R., Ming J. (2019). Comparison of phenolic profiles and antioxidant activities in skins and pulps of eleven grape cultivars (*Vitis vinifera* L.). J. Integr. Agric..

[71] Gajera H.P., Katakpara Z.A., Patel S.V., Golakiya B.A. (2016). Antioxidant defense response induced by *Trichoderma viride* against *Aspergillus niger* Van Tieghem causing collar rot in groundnut (*Arachis hypogaea* L.). Microb. Pathog..

[72] Masuo S., Osada L., Zhou S., Fujita T., Takaya N. (2015). *Aspergillus oryzae* pathways that convert phenylalanine into the flavor volatile 2-phenylethanol. Fungal Genet. Biol..

[73] Hazelwood L.A., Daran J.M., Van Maris A.J.A., Pronk J.T., Dickinson J.R. (2008). The Ehrlich pathway for fusel alcohol production: A century of research on *Saccharomyces cerevisiae* metabolism. Appl. Environ. Microbiol..

[74] Poole P. (2017). Shining a light on the dark world of plant root–microbe interactions. Proc. Natl. Acad. Sci. USA.

[75] Ho J., Prosser R., Hasani M., Chen H., Skanes B., Lubitz W.D., Warriner K. (2020). Degradation of chlorpyrifos and inactivation of *Escherichia coli* O157:H7 and *Aspergillus niger* on apples using an advanced oxidation process. Food Control.

