# Supplementary material for: Mapping Aspergillus niger Metabolite Biomarkers for In Situ and Early Evaluation of Table Grapes Contamination
Source: Foods. 2021 Nov 19;10(11):2870. doi: 10.3390/foods10112870 (PMC8624196; doi:10.3390/foods10112870)
Supplement: Supplementary file 1 [file foods-10-02870-s001.zip › Supplementary Information - Figure S1.pdf]

foods

MDPI

Supplementary Information

Mapping *Aspergillus niger* Metabolite Biomarkers for In Situ and Early Evaluation of Table Grapes Contamination

João Raul Belinato [1], Carina Pedrosa Costa [2], Adelaide Almeida [3], Silvia M. Rocha [2,*] and Fabio Augusto [1,*]

[1] Institute of Chemistry, University of Campinas and National Institute of Science and Technology in Bioanalysis (INCTBio), Brazil.
[2] Department of Chemistry & LAQV-REQUIMTE, University of Aveiro, 3810-193 Aveiro, Portugal.
[3] Department of Biology & CESAM, University of Aveiro, 3810-193 Aveiro, Portugal.

* Correspondence: smrocha@ua.pt and faugusto@unicamp.br

foods

MDPI

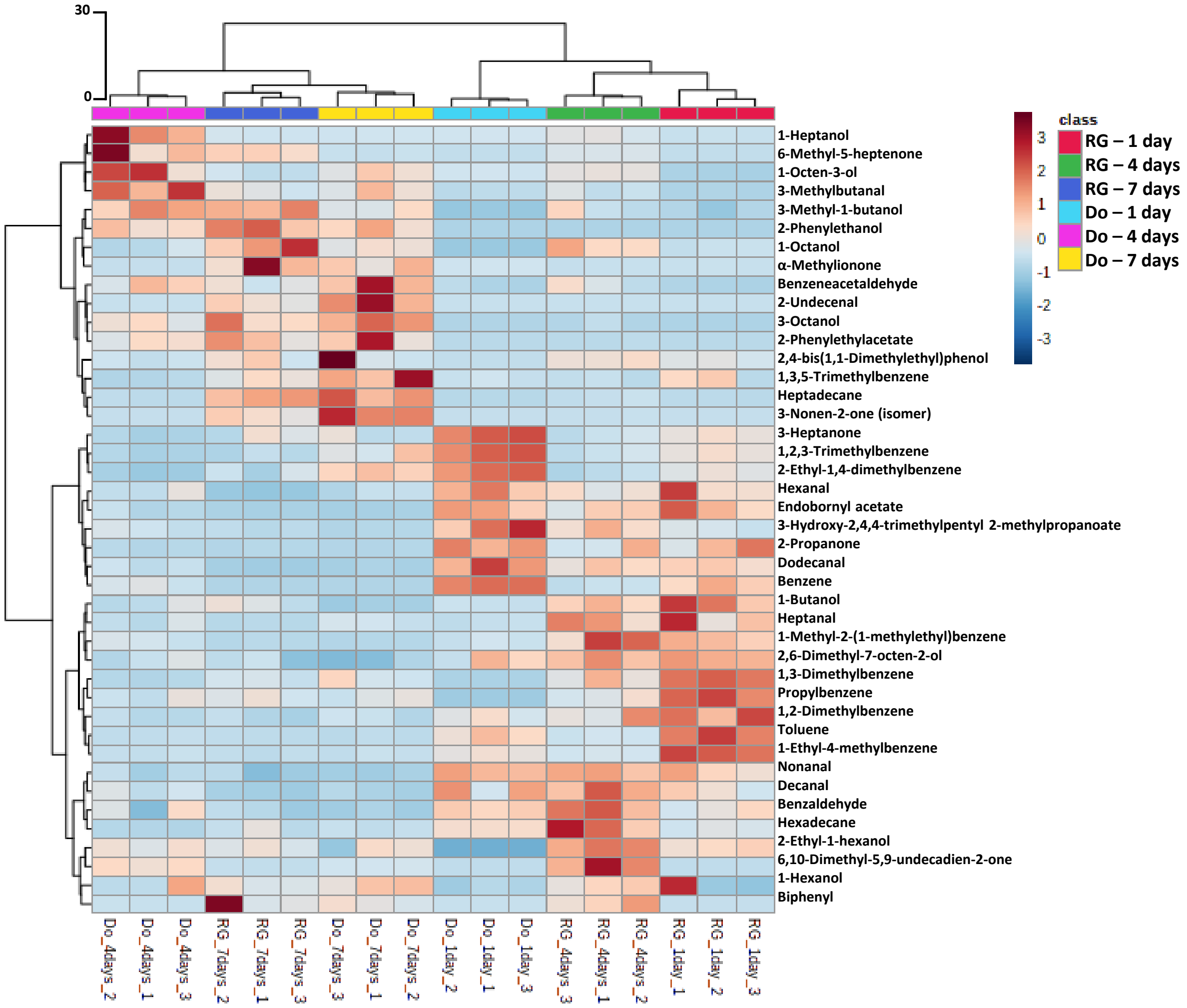

Figure S1. Dendrogram and heatmap representation of the 42 metabolites putatively identified from red (RG) and white (Do) tables grapes contaminated with *A. niger*, determined by in-situ HS-SPME/GC×GC-ToFMS, over 7 days, which illustrates the formation of two main clusters related to the two grape varieties, within each one there is an organization of the samples as a function of the growth time.
